# Initial Description of the Genome of *Aeluropus littoralis*, a Halophile Grass

**DOI:** 10.3389/fpls.2022.906462

**Published:** 2022-07-11

**Authors:** Seyyed Hamidreza Hashemi-Petroudi, Mozhdeh Arab, Behnaz Dolatabadi, Yi-Tzu Kuo, Mariana Alejandra Baez, Axel Himmelbach, Ghorbanali Nematzadeh, Seyed Ali Mohammad Mirmohammady Maibody, Thomas Schmutzer, Michael Mälzer, Thomas Altmann, Markus Kuhlmann

**Affiliations:** ^1^Genetics and Agricultural Biotechnology Institute of Tabarestan (GABIT), Sari Agricultural Sciences and Natural Resources University, Sari, Iran; ^2^RG Heterosis, Leibniz Institute of Plant Genetics and Crop Plant Research (IPK), Gatersleben, Germany; ^3^Research Group Chromosome Structure and Function, Leibniz Institute of Plant Genetics and Crop Plant Research (IPK), Gatersleben, Germany; ^4^Research Group Genomics of Genetic Resources Cereals Research, Leibniz Institute of Plant Genetics and Crop Plant Research (IPK), Gatersleben, Germany; ^5^Department of Agronomy and Plant Breeding, College of Agriculture, Isfahan University of Technology, Isfahan, Iran; ^6^Institute of Agricultural and Nutritional Sciences, RG Biometrics and Agroinformatics, Martin-Luther-University Halle-Wittenberg, Halle (Saale), Germany; ^7^RG Structural Cell Biology, Leibniz Institute of Plant Genetics and Crop Plant Research (IPK), Gatersleben, Germany

**Keywords:** *Aeluropus littoralis*, halophile, genome, genome size, repetitive elements, DREB

## Abstract

The use of wild plant species or their halophytic relatives has been considered in plant breeding programs to improve salt and drought tolerance in crop plants. *Aeluropus littoralis* serves as halophyte model for identification and isolation of novel stress adaptation genes. *A. littoralis, a* perennial monocot grass, grows in damp or arid areas, often salt-impregnated places and wasteland in cultivated areas, can survive periodically high water salinity, and tolerate high salt concentrations in the soil up to 1,100 mM sodium chloride. Therefore, it serves as valuable genetic resource to understand molecular mechanisms of stress-responses in monocots. The knowledge can potentially be used for improving tolerance to abiotic stresses in economically important crops. Several morphological, anatomical, ecological, and physiological traits of *A*. *littoralis* have been investigated so far. After watering with salt water the grass is able to excrete salt *via* its salt glands. Meanwhile, a number of ESTs (expressed sequence tag), genes and promoters induced by the salt and drought stresses were isolated, sequenced and annotated at a molecular level. Transfer of stress related genes to other species resulted in enhanced stress resistance. Here we describe the genome sequence and structure of *A. littoralis* analyzed by whole genome sequencing and histological analysis. The chromosome number was determined to be 20 (2*n* = 2x = 20). The genome size was calculated to be 354 Mb. This genomic information provided here, will support the functional investigation and application of novel genes improving salt stress resistance in crop plants. The utility of the sequence information is exemplified by the analysis of the DREB-transcription factor family.

## Introduction

**FIGURE 1:**
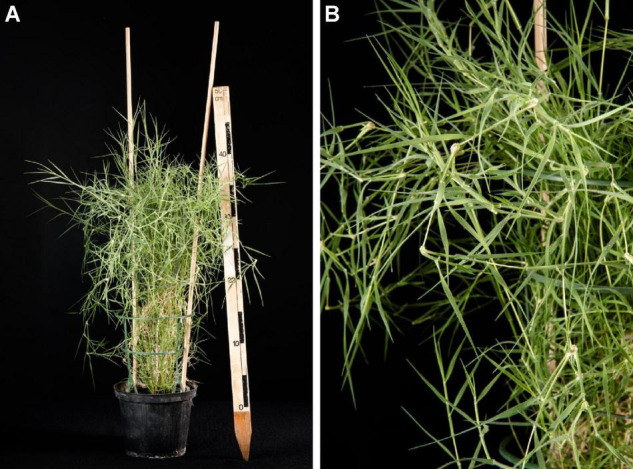
**(A)** *Aeluropus littoralis* cultivated in pots under greenhouse condition. **(B)** Enlargement of leaves. Black and white box on the ruler represents 5 cm.

The use of wild plant species or their halophytic relatives has been considered in plant breeding programs to improve salt and drought tolerance in crop plants (Ben-Saad et al., 2012). *Aeluropus littoralis* (Watson et al., 1986) is a monocot belonging to the Gramineae (Poaceae) family, subfamily *Chloridoideae* (Peterson et al., 2010), also referred to as “Indian walnut” and first described 1764 by Antoine Gouan (Figure 1A). It serves as halophyte model for identification and isolation of novel stress adaptation genes. This species is described as perennial grass with an estimated small haploid genome of 349–8,232 Mbp (Zonneveld et al., 2005; Zouari et al., 2007; Modarresi et al., 2012) and it possess a C_4_ mechanism for carbon fixation (Wang, 2004; Barhoumi et al., 2007a) with Kranz anatomy and a Mediterranean, Irano-Turanian Chlorotype (Frey and Kurschner, 1983) isolated from their natural habitat. An early study (Shomeril and Waisel, 1973) described the influence of salt, shifting the C_3_ metabolism toward C_4_ metabolism. Such change was also reported lately for other halophile plants (Bose et al., 2017), but is still debated as such mechanism was not described for any other Poacea yet. A salt induced change from C_3_ to CAM metabolismus is also a frequently observed strategy of plants to cope with high levels of salt (Winter and Holtum, 2014; Brilhaus et al., 2016).

*Aeluropus littoralis* is widely distributed and can be found in Northern Africa, in temperate and tropical areas of Asia, southern and south eastern part of Europe. *A. littoralis* grows in damp or arid areas, often salt-impregnated places and wasteland in cultivated areas (Saad et al., 2011). *A. littoralis* is primarily found in desert regions and regions with high soil salinity due to flooding and can survive where the water salinity is periodically high (Mesléard et al., 1993) and tolerate extreme high salt concentrations in the soil up to 1,100 mM sodium chloride (Barhoumi et al., 2007a). The plant is able to secrete salt *via* its salt glands leading to formation of salt crystals on the leaf surface (Barhoumi et al., 2007a,b). From an economic point of view the plants are important for reclaiming salinized agricultural and rangeland, they are used for sand fixation and grow on pastures. Particularly in developing countries (Gulzar et al., 2003) they are extensively used as fodder crop. The grass is also capable of vegetative reproduction through rhizome growth after monsoon rains and can produce numerous flowers and seeds from April to October (Gulzar and Khan, 2001). Due to its high salt tolerance *A. littoralis* serves as valuable genetic resource to understand molecular mechanisms of stress-responses in monocots (Azri et al., 2016). This knowledge can potentially be used for improving tolerance to abiotic stresses in economically important crops (Saad et al., 2010). Several morphological, anatomical, ecological, and physiological traits of *A*. *littoralis* have been investigated so far (Barhoumi et al., 2007a,^2008^; Rezvani et al., 2012). The transfer of stress related genes to other species resulted in enhanced stress resistance (Ben Saad et al., 2010, 2012; Liu et al., 2014; Ben Romdhane et al., 2017; Ghneim-Herrera et al., 2017).

This species can grow to a height of 30 cm. The leaves are distichous and leaf sheaths are longer than adjacent culm internodes (Figure 1B). The leaf blades are 1–5 cm in length and 1–2 mm wide. They appear stiff and glaucous, while the leaf surface is ribbed. The inflorescence is composed of two to twelve racemes borne along a central axis. The central inflorescence axis is 1–4 cm long and the solitary spikelets are packed on the broadside of the rachis (Liu et al., 2005). They are on a bilateral false spike and can be termed two-rowed. The spikelets comprise of six to nine fertile florets with diminished florets at the apex.

As several contradicting genome features are found in the literature (genome size, chromosome number, presence of B chromosomes). Cytogenetically, for the chromosome number of *A. littoralis* a variation between 2*n* = 2x = 14 and 2*n* = 2x = 20 was previously reported (Modarresi et al., 2012). Likewise, the genome size varies from 342 Mb (Zouari et al., 2007; Modarresi et al., 2012) to 8,215 Mbp (Zonneveld et al., 2005). These points were revisited here in addition to the genome sequence information.

In order to exemplify the utility of the available sequence information, the DREB-transcription factor gene family in *Aeluropus littoralis* was investigated and results presented. The DREB transcription factor subfamily belongs to the APETALA2/ETHYLENE-RESPONSIVE FACTOR (AP2/ERF) superfamily of transcriptional regulators. Members of this family share a specific variant of the AP2 domain including a valine (Val14) and glutamine (Glu19) residue, as well as the YRG motif and the RAYD motif (Sakuma et al., 2002). Proteins encoded by this gene family bind to a 9-bp conserved sequence (TACCGACAT) defined as dehydration-responsive element (DRE) (Huang et al., 2019). The role of this transcription factor family in the integration of salinity stress and drought stress was presented in Dubouzet et al. (2003) in *Oryza sativa*, a relative of *A. littoralis* from the poacea family.

## Materials and Methods

### Plant Material

*Aeluropus littoralis* seeds were collected from the Isfahan province in Iran and plants were cultivated at IPK Gatersleben (Germany) and Sari Agricultural Sciences and Natural Resources University (Iran). A specimen of the analyzed plants was deposited at the herbarium GAT under voucher number 70486. Sterilized seeds were plated on full strength MS medium (Murashige and Skoog, 1962) with vitamins, 3% sucrose and 0.7% agar (pH 5.8). The cultures were incubated in a germinator at 25 ± 2°C with 16 h light/8 h dark photoperiod at 100 μmol m^–2^ s^–1^ photon flux density using cool-white fluorescent light. Two weeks after germination, the seedlings were transferred to hydroponic culture containing Hoagland’s solution (Arnon and Hoagland, 1939). Hoagland’s nutrient solution comprised 3 mM KNO_3_, 2 mM Ca(NO_3_), 2.1 mM NH_4_H_2_PO_4_, 0.5 mM MgSO_4_, 1 μM KCl, 25 μM H_3_BO_3_, 2 μM MnSO_4_, 2 μM ZnSO_4_, 0.1 μM CuSO_4_, 0.1 μM (NH_4_)_6_Mo_7_O_24_ and 20 μM Fe(Na) EDTA, in demineralized H_2_O buffered with 2 mM 2-(*N*-morpholino) ethanesulphonic acid, pH 5.5, adjusted using KOH. Transferred plants were grown in a phytochamber at approximately 240 μmol m^–2^s^–1^ under longday photoperiodic conditions (16 h light, 22°C/8 h dark, 18°C) at 70% relative humidity.

For salt stress treatments soil grown plants (pots 12 cm diameter) were watered with 1 m NaCl once per week.

### Light and Transmission Electron Microscopy

*Aeluropus littoralis* leaves of three biological replicates of plants grown under controlled conditions or exposed to salt stress were used for comparative histological and ultrastructural analyses. Cuttings of a size of 1 mm × 2 mm from the central part of fully developed leaves were used for combined conventional and microwave assisted chemical fixation, substitution and resin embedding as defined in the given protocol (Supplementary Table 1). Sectioning, histological staining, light and transmission electron microscopy analysis was performed as described (Daghma et al., 2011).

### Chromosome Preparation and Fluorescence *in situ* Hybridization

Mitotic chromosomes were prepared from root tips, which were pretreated in ice water for 24 h to accumulate synchronized cells at metaphase, fixed in Carnoy’s fixative [ethanol and glacial acetic acid, 3:1 (v/v)] at room temperature for 20 h, and kept in 70% ethanol at 20°C for later use. Fixed roots were digested in an enzyme mixture (2% cellulose, 2% pectinase, 2% pectolyase in citrate buffer containing 0.01 M sodium citrate dihydrate and 0.01 M citric acid) at 37°C for 30–40 min. Cell suspension from root meristems in Carnoy’s fixative was dropped onto slides on a hot plate at 50°C, slides were further fixed in the fixative for 1 min, air-dried, and kept at 4°C.

The clones pTa71 and pAt T4 were labeled with fluorophore ATTO550 and ATTO488, respectively, using nick translation labeling kit (Jena Bioscience) to detect 45S rDNA loci and Arabidopsis-type telomeres. Fluorescence *in situ* hybridization was performed as described in Aliyeva-Schnorr et al. (2015) with pretreatment for 10 min in 45% acetic acid at room temperature, followed by 0.1% pepsine in 0.01N HCl at 37°C. Slides were supplied with the hybridization mix (50% (v/v) formamide, 10% (w/v) dextran sulfate, 2 × SSC, and 3 ng/μl of telomere probe) and denatured at 75°C for 2 min. After stringency wash in 2 × SSC at 57°C for 20 min, chromosomes were counterstained with 4′,6-diamidino-2-phenylindoline (DAPI). Images were captured using an epifluorescence microscope BX61 (Olympus) equipped with a cooled CCD camera (Orca ER, Hamamatsu) and pseudocolored with Adobe Photoshop.

### Estimation of Nuclear Genome Size

For estimation of nuclear genome size by flow cytometry, approximately 10 mm^2^ of leaf tissue from individuals of *Aeluropus littoralis* plants was chopped with a sharp razor blade together with roughly 5 mm^2^ of leaf material of *Raphanus sativus* cv. “Voran” (Genebank Gatersleben accession number: RA 34; 2C = 1.11 pg) as internal reference standard (Schmidt-Lebuhn et al., 2008) in a Petri dish containing 1 ml Galbraith nuclei isolation buffer (Galbraith et al., 1983) supplemented with 1% PVP- 25, 0.1% Triton X-100, *DNase*-free *RNase* (50 μg/ml). The nuclei suspension was filtered through a 35-μm mesh cell strainer cap to remove large fragments and stored on ice until measurement. The relative fluorescence intensities of 7,000–10,000 events (nuclei) per sample were measured using a CyFLow Space flow cytometer (Sysmex-Partec, Germany) quipped with a 30 mW green solid state laser (532 nm). The absolute DNA amounts of samples were calculated based on the values of the G1 peak means.

### Extraction of Genomic DNA

DNA of *A. littoralis* was extracted according to Dellaporta procedures (Dellaporta et al., 1983). The quality and quantity of the extracted DNA were controlled by measuring absorbance at 260/280 nm using a NanoDrop spectrophotometer (Biochrom WPA Biowave II, United Kingdom). Further, the purity and integrity of DNA were tested by running on 0.7% agarose gel electrophoresis.

### Illumina Sequencing and Sequence Data Pre-processing

Library preparation (Illumina TruSeq DNA Sample Prep Kit) and sequencing by synthesis using the Illumina HiSeq2500 device involved standard protocols from the manufacturer (Illumina, Inc., San Diego, CA, United States). The library was quantified by qPCR (Mascher et al., 2013) and sequenced using the rapid run mode (on-board cluster generation, paired-end, 2 × 101 cycles. In total, 125,600,517 Illumina paired end reads were produced having a total output of residues of 42.5 Gb. Prior to the assembly process reads were quality trimmed using clc_quality_trim with a minimal cut-off threshold of Q30 and default settings on remaining parameters. 85.6% of reads and 83.77% of residues passed this initial pre-processing. Subsequently, the quality of the sequence data was checked using fastQC^1^. After this quality enrichment a genome coverage of 62-fold was reached.

### *De novo* Assembly Construction

Our *A. littoralis de novo* sequence was constructed using CLC assembly cell version 4.3 and the quality trimmed WGS data. The *de novo* assembly pipeline was applied with automatic detection of best parameters by CLC assembly cell. In accordance with good practice, all contigs below a length threshold of 200 bp were removed. For purification of the constructed assembly we checked our constructed contigs for contamination by *E. coli* using BLAST + (Camacho et al., 2009). As parameter settings we used a sequence identity of 60% and a word size of 28. Critical contigs were fully removed if the BLASTN analysis resulted in a hit with length > 500 bp. For smaller contigs we reduced the minimal length of a hit to 200 bp, while at the same time at least 10% of the length of the contig is identified as *E. coli* contamination. From the remaining sequences we removed contigs in case a bacterial origin was detected within the BLASTN analysis against the NCBI non-redundant nucleotide database nt. In addition, we filtered for contigs having a length of 500 bp. The descriptive statistics of both datasets (200 bp and 500 bp) are given in Table 1. The list of all contigs is available at https://doi.ipk-gatersleben.de/DOI/ca99c593-ffdd-4d49-8eab-f1c891953776/d5b041b5-b2c1-4696-bc7c-bc5a32a0c7ec/2/1847940088.

**TABLE 1:** Descriptive statistics of WGS assembly.

Parameter	*A. littoralis* WGS assembly	*A. littoralis* WGS assembly (> 500 bp)
Number of contigs	182,747	113,845
Total number of bases	300,381,201	277,993,896
Minimal contigs length	200	500
Maximal contigs length	69,774	69,774
N25 contig length (bp)	7,574	8,048
N50 contig length (bp)	3,649	4,074
N75 contig length (bp)	1,477	1,869
GC content	0.44	0.44

### Gene Prediction and Annotation

We used the purified WGS assembly without a threshold on contig sizes to predict gene models. Gene prediction was done with GeMoMa (Keilwagen et al., 2016) using gene models of *Brachypodium distachyon* (Brachypodium_distachyon_v3.0, INSDC Assembly GCA_000005505.4, Feb 2018), *Oryza sativa* (IRGSP-1.0, INSDC Assembly GCA_001433935.1, Oct 2015) and *Sorghum bicolor* (Sorghum_bicolor_NCBIv3, INSDC Assembly GCA_000003195.3, Apr 2017) downloaded from Ensembl Plants (Bolser et al., 2017). In total, 15,916 gene models were predicted in 12,130 different contigs. For all detected gene models CDS (FASTA), protein sequence (FASTA) and genomic positions (GFF) are provided. We further investigated these datasets performing a gene annotation with AHRD version 2.0^2^ using UniProt, trembl and TAIR10 (downloaded January 4th 2016). For 13,921 genes (87.5%, Supplementary Table 2) a functional annotation could be assigned (Supplementary Table 3). The complete dataset of gene models and functional annotation is available for download. The coding sequences of all annotated genes are available at https://doi.ipk-gatersleben.de/DOI/ac423f10-971e-481e-bcab-6ac261e27f5c/15d455e1-da91-4e78-82d8-7c7607cb05b9/2/1847940088 (provisional DOI). DOIs of datasets released in this manuscript were constructed using the e!DAL system (Arend et al., 2014).

### Genome Repeat Fraction Analysis

The repetitive fraction analysis was performed with 89 Mbp of reads of the total genomic DNA (0.26x genome coverage). Quality trimmed reads were grouped with the graph-based clustering algorithm based on sequence similarity, implemented in the RepeatExplorer pipeline (Novak et al., 2013). The paired-end reads clustering was performed with a minimum overlap of 55% and a similarity of 90%. Three independent analyses were performed, using a different dataset of reads of the same sequencing, to confirm the proportions of each cluster within the total genome. Repeat annotation and classification was performed for those clusters with an abundance of at least > 0.01%. For basic repeat classification, protein domains were identified using the tool “Find RT Domains” within RepeatExplorer (Novak et al., 2013). Searches for sequence similarity, using different databases (RepeatMasker and GenBank) were performed and graph layouts of individual clusters were examined using the SeqGrapheR program (Novak et al., 2013). Satellite DNAs were identified based on the TAREAN tool implemented in the pipeline, graph layouts and further examined using DOTTER (Sonnhammer and Durbin, 1995).

### Analysis of DREB Gene Family

A total of 57 DREB proteins from rice (Oryza sativa) and Arabidopsis (Arabidopsis thaliana) were retrieved from the Michigan State University (MSU) rice genome annotation database^3^, and the Arabidopsis information resource (TAIR) database^4^, respectively.

These data were utilized to find DREB genes (*AlDREB*) in *A. littoralis* genomes as the query. AlDREB protein sequences were searched using two approaches. The first technique employed a tBlastn to search against *A. littoralis* genome sequences, while the second employed BLASTP (E-value 1e5) against *A. littoralis* protein sequence. Genomic, protein, and CDS (coding DNA sequence) sequences of *AlDREB* gene family have been identified. To verify the search results, we inspected and analyzed all candidate sequences using Pfam^5^ (El-Gebali et al., 2019), InterProScan^6^ (Jones et al., 2014), and SMART^7^ (Letunic and Bork, 2018) tools. The ProtParam Tool^8^ was used to determine the theoretical isoelectric point (pI) and molecular weight (MW) of the discovered proteins. The MEME website^9^ was used to find conserved motifs in AlDREB protein sequences (Bailey et al., 2009).

Protein motifs and gene structure were visualized by using TBtools software (Chen et al., 2018). Inferring phylogenetic relationships was done with MEGA7.0 software (Kumar et al., 2016) and the Maximum Likelihood (ML) approach based on the LG model.

## Results

To validate the chromosome number of the used material we performed chromosome countings. Furthermore, we analyzed the size, structure and composition of the *Aeluropus littoralis* genome. Using a high-throughput sequencing approach on the genome sequence a first assembly of the genome sequence is presented here. Meanwhile, a number of ESTs (expressed sequence tag), genes and promoters induced by the salt and drought stresses were isolated, sequenced and annotated at a molecular level (Saad et al., 2011; Ben-Saad et al., 2012). Here we describe the genome sequence and structure of *A. littoralis* analyzed by whole genome sequencing and histological analysis. This genomic information will support the functional investigation and application of novel genes improving stress resistance in crop plants.

**FIGURE 2:**
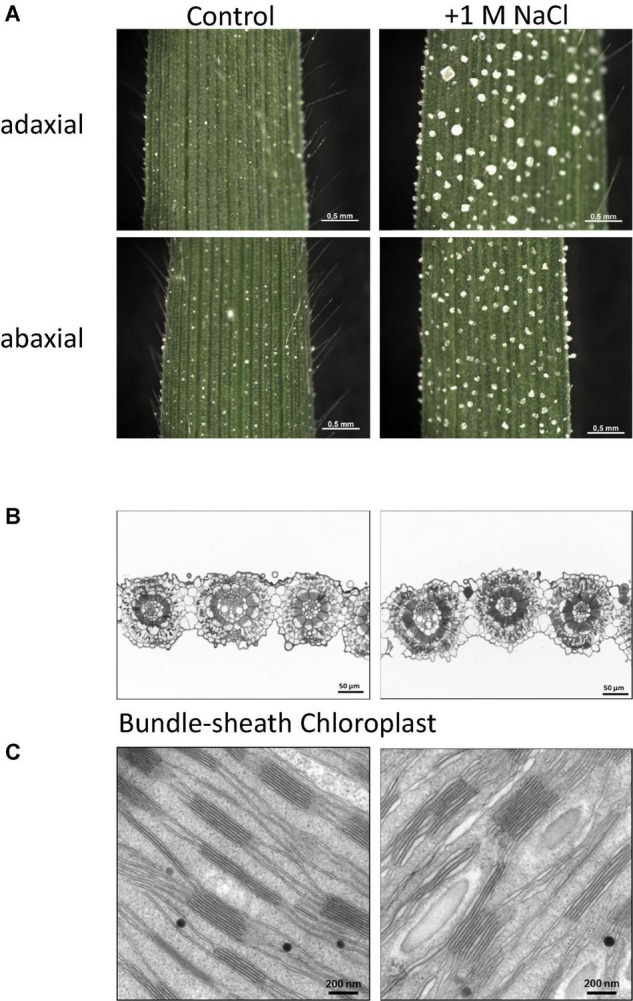
**(A)** Leaf surface of young leaved developed in pots under greenhouse conditions at different watering regimes. Left: control (tap water) and right: salt watering (1 M NaCl). Shown are adaxial and abaxial sides of the leaves including formed salt crystals at salt watering condition. **(B)** Microscopic section of leaves after methylblue/azur II stain showing Kranz anatomy, typical indication for C4 type plant. Bundle sheath cells appear darker at salt treatment. **(C)** SEM analysis of thylakoid structure in bundle sheath cell chloroplasts. Grana staples are larger and show spaces between layers at salt treatment.

For the genome analysis *A. littoralis* leaf tissue grown under greenhouse conditions, was used. Seeds were collected from Isfahan province in central Iran. This region experiences a moderate and dry climate with temperature ranging between 10.6°C. and 40.6°C. The annual rainfall in this region on an average has been reported as 116.9 mm and can be considered super arid (desert) climate. Salt stress condition was applied by watering with 1 M NaCl solution instead of tap water under greenhouse conditions. When the plants are exposed to high amounts of salt water (e.g., 1 M NaCl) they start to develop salt glands and extrude the salt in crystals on the leaves (Barhoumi et al., 2007a,b). Figure 2A shows the salt crystals formed on the leaf surface. Crystals of cubic shape are formed on the adaxial and abaxial side of the leaf at the salt glands. As very early reports indicate a shift from C_3_ to C_4_ carbon fixation mechanism, we investigated the leaf structure under control and salt conditions. New leaves developed under control and salt watering conditions were analyzed. Under both conditions a Kranz anatomy structure (Figure 2B) was found: the enlarged bundle sheath (BS) cells surround the veins and the BS cells are then surrounded by mesophyll (M) cells. Interestingly, when stained with methyleneblue/azur II (Richardson et al., 1960) the bundle sheath cells appear darker under salt stress conditions. This might indicate an accumulation of acidic components under salt stress. The bundle sheath cells appear more closed and filled with thylakoids. The ultrastructural analysis shows an increase of thylakoid staples and wider spacing of the staples (Figure 2C), leading to a higher volume. Whether this indicates a higher activity or a disintegration of chloroplasts coupled with repair mechanism remains to be solved.

### Size and Structure of the *Aeluropus littoralis* Genome

**FIGURE 3:**
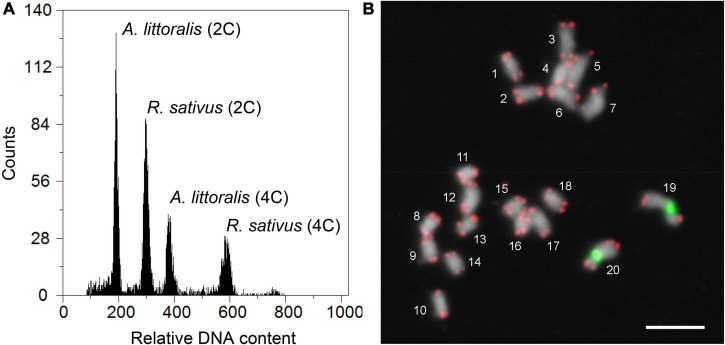
Genome size estimation and chromosome counting of *Aeluropus littoralis*. **(A)** Measurement of *A. littoralis* genome size by flow cytometry. Based on the 2C-value of 1.11 pg for the internal reference *Raphanus sativus*, the average DNA content of diploid *A. littoralis* was found to be 0.724 ± 0.01 pg/2C (354 Mbp/1C). The flow cytometric histogram represents the relative fluorescence intensity of leaf nuclei between *A. littoralis* and *R. sativus*. **(B)** FISH mapping of telomere repeat (red) and 45S rDNA (green) clearly indicating that the chromosome number of *A. littoralis* is 2*n* = 2x = 20. Chromosomes were counterstained with DAPI. Scale bar = 5 μm.

In order to support our whole genome sequencing data, we were addressing the question of the *A. littoralis* genome structure. A chromosome number between 2*n* = 2x = 14 and 2*n* = 2x = 20 was previously reported for *A. littoralis* and deposited in different (Zouari et al., 2007; Modarresi et al., 2012) chromosome databases (Rice et al., 2015). Likewise, descriptions of the genome size vary from 342 Mb (Zouari et al., 2007; Modarresi et al., 2012) to 8,215 Mbp (Zonneveld et al., 2005). Therefore the nuclear genome size was estimated by flow cytometry (Figure 3A) using *Raphanus sativus* cv. “Voran” (Genebank Gatersleben accession number: RA 34; 2C = 1.11 pg) as reference (Schmidt-Lebuhn et al., 2008). Relative fluorescence intensities of around 7,000–10,000 events (nuclei) per sample were measured and the absolute DNA amounts of samples were calculated based on the values of the G1 peak means. The DNA content of the diploid *A. littoralis* was estimated to be 0.724 ± 0.01 pg/2C (354 Mbp/1C) and is therewith only slightly bigger than reported previously (Wang, 2004). To validate the chromosome number, karyotyping was performed on mitotic chromosome spreads. The chromosome number was determined to be 20 (2*n* = 2x = 20, Figure 3B). However, occasionally also metaphases with 21 or 22 chromatin units were found. To analyze if these additional chromatin units resulted from satellites being located distally from the corresponding chromosomes or were indeed B-chromosomes, as it was sporadically reported (Liu et al., 2005), fluorescence *in situ* hybridization (FISH) with the *Arabibidopsis*-type telomere repeat and 45S rDNA was performed (Figure 3B). The resulting hybridization pattern clearly indicates that the increased number of chromatin units are a consequence of extended nucleolus organizing regions (NORs).

### Whole Genome Sequencing and *de novo* Assembly

The genome of *A. littoralis* was sequenced using a whole-genome sequencing approach (WGS) on Illumina’s HiSeq 2500 system. In total, 125 million paired end (PE) reads were produced, reaching genome coverage after quality trimming of approximately 62-fold. This data was sufficient to perform a *de novo* assembly to construct the first available genomic reference for the *A. littoralis* species. The constructed genome sequence reached a total size of ∼300 Mbp, which corresponds to 84.7% of 354 Mbp estimated here or 87.7% of the previously estimated genome size of 342 Mbp (Zouari et al., 2007). The assembly consists of 182,747 contigs with a N50 contig length of 3.6 kb. The constructed genomic resource was used for gene prediction and was complemented by a functional annotation of genes. In total, 15,916 gene models were predicted for *A. littoralis* and 87.5% of them could be assigned a function based on sequence similarity to known genes (Table 1 and Supplementary Figure 1).

### Repetitive Fraction in the *Aeluropus littoralis* Genome

**FIGURE 4:**
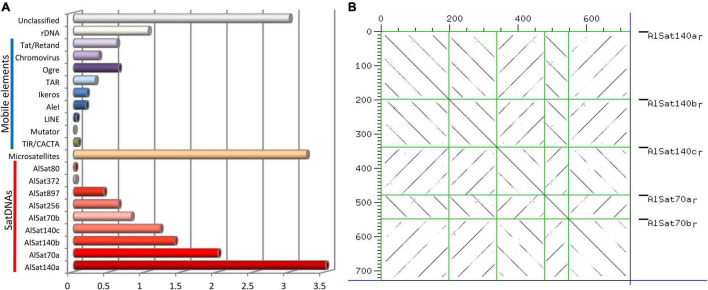
**(A)** *Aeluropus littoralis* genome repetitive composition, satDNAs are the most abundant repetitive element within the genome. **(B)** Dot-plot showing the sequences similarity between the different variants of the same satellite family, AlSat140.

In order to characterize the repetitive DNA fraction of *A. littoralis* the reads from the paired end WGS were used. Reads, comprising in total 0.26-fold genome coverage, were grouped based on sequence similarity into 33,385 clusters containing from 2 to 21,265 reads. Clusters included 32% of all reads, with the major 282 clusters representing at least 0.01% of the genome each. The cluster analysis revealed that 21.69% of the *A. littoralis* genome is composed of repetitive elements with nine satellite DNA families (satDNAs), nine transposable elements families (LTR-retrotransposons and LINE), two DNA transposons families (CACTA-like and Mutator-like), ribosomal DNA (35S and 5S) and microsatellites (Table 2 and Figure 4A). The most abundant repetitive families were satDNAs, ∼11% of the genome, with the five largest clusters being part of the superfamily AlSat140. Within this superfamily five variants could be identified: AlSat140a, AlSat140b, AlSat140c, AlSat70a and AlSat70b, with 85–96% similarity of the monomer sequence (Figure 4B). Beside these satDNAs, four other satDNAs families were identified in the genome: AlSat256, AlSat897, AlSat372 and AlSat80, with 0.62%, 0.42%, 0.03% and 0.02% of the genome, respectively. The LTR-like retrotransposons constituted 2.22% of the genome, with the Ty3/Gypsy superfamily exceeding 2.4 fold the genome proportion of the Ty1/Copia superfamily. Within the former, Tat/Retand, Tat/Ogre and Chromovirus were the only highly abundant lineages. Within Ty1/Copia retrotransposons the *Ale*I, Ikeros and TAR lineages were identified, with the last being most abundant. Microsatellites were identified in several different clusters comprising 3.23% of the genome (Table 2 and Figure 4A).

**TABLE 2:** Genome proportion of the repetitive elements identified in *A. littoralis*.

	Repetitive element		Total genome%
	Satellite	AlSat140a	3.98
		AlSat70a	2.29
		AlSat140b	1.60
		AlSat140c	1.33
		AlSat256	0.71
		AlSat897	0.48
		AlSat71	0.80
		AlSat372	0.03
		AlSat80	0.02
	Microsatellites		3.23
Class I			
	LTR-Ty3/Gypsy	Tat/Ogre	0.62
		Chromo/Tekay	0.32
		Chromo/Reina	0.01
		Chromo/CRM	0.02
		Tat/Retand	0.60
	LTR-Ty1/Copia	*Ale*I	0.17
		Ikeros	0.18
		TAR	0.30
	LINE		0.04
Class II			
	DNA Transposons	CACTA	0.06
		Mutator	0.01
	rDNA		1.04
	Unclassified		3.84
	Total		21.69

### Analysis of DREB Subgene Family in Aeluropus to Exemplify the Utility of the Available Genome Data

**FIGURE 5:**
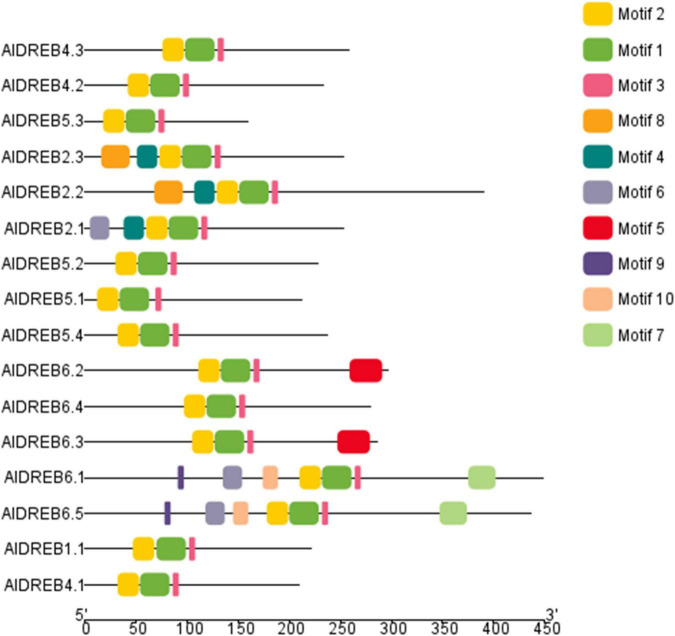
Structure of identified DREB related proteins in *Aeluropus littoralis*. Based on the available sequence information 16 DREB gens could be identified encoding proteins with DREB associated motifs. Detailed motif information is given in the Supplementary Table 5. Motif 1, 2 and 3 depict the AP2-superfamily defining motifs.

**FIGURE 6:**
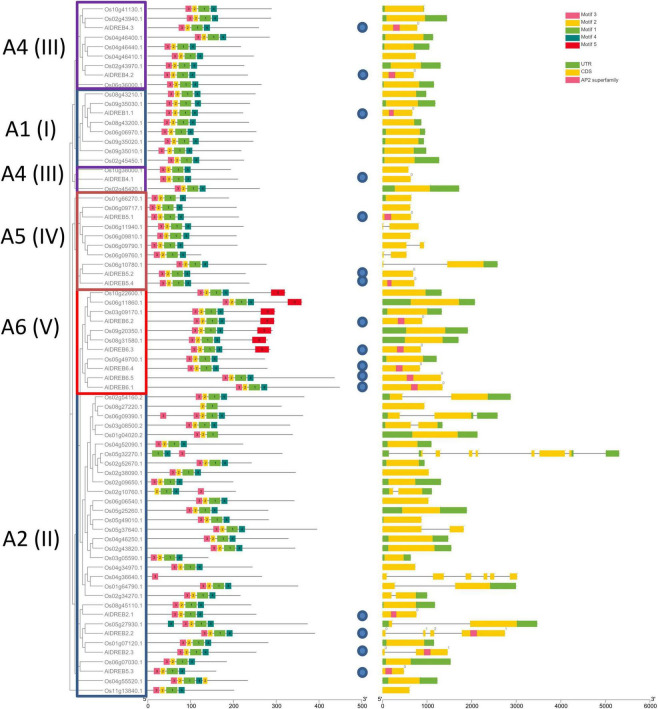
Phylogenetic structure of *Aeluropus littoralis* DREB-proteins. Phylogenetic comparison *of Aeluropus littoralis* DREB-proteins (blue circles) with Oryza sativa DREB-proteins. Individual subfamilies (Sakuma et al., 2002) A1-A6 (I-V) are outlined by boxes and specific sequence motifs are given in the legends. (The respective AA sequence of the motifs can be found in Supplementary Table 4). On the right side the schematic information is given of the related genic sequence.

As proof of concept for a possible utilization of our genome sequence data the dehydration responsive element-binding (DREB) transcription factor family in Aeluropus was investigated. Members of this family share a specific variant of the AP2 domain including a valine (Val14) and glutamine (Glu19) residue, as well as the YRG motif and the RAYD motif (Sakuma et al., 2002). The name giving AP2-domain is indicated in Figure 5 by the motif combination 3-2-1-4. The YRG domain is depicted as motif 3 (pink) and the RAYD domain as motif 1 (green). The genome wide analysis of the DREB-subfamily in Aeluropus was performed as described (Huang et al., 2019). In total, 16 non-redundant genes (Supplementary Table 4) encoding proteins containing DREB-related motifs (Supplementary Table 5) were identified from the genomic sequences of *A. littoralis* (Figure 5). All of these proteins contain the family determining motifs 1–4. Figure 6 shows the phylogenetic comparison of the *A. littoralis* sequences in comparison to the available information for rice (*Oryza sativa*) derived DREB sequences (Dubouzet et al., 2003). The classification is based on the encoded domain structure and the nomenclature defined by Sakuma et al. (2002). In comparison with the rice DREB gene family sequence (Chai et al., 2020), the identified Aeluropus sequences can be grouped into the subfamilies as depicted in Figure 6. In order to extend this analysis to the dicotyledonous model plants *Arabidopsis thaliana*, the available sequence information was retrieved and a combined phylogenetic tree generated (Supplementary Figure 2). The identified sequences represent at least one member of each subfamily. The subfamily A2 (II), usually is the subfamily with the highest variation and largest number of representatives. In our dataset the subfamily A2 (II) is represented by four members. An unusual high number of five genes in the Aeluropus genome can be assigned to subfamily A6 (V). Members of this subfamily (like RAP2.4) are involved in stress-specific changes of leaf morphology (Yang et al., 2020).

## Discussion

Based on our results, summarized in Table 3, the chromosome number of *A. littoralis* is 20 (2*n* = 2x = 20). As pointed out before, the available information from prior published data (Zouari et al., 2007; Modarresi et al., 2012; Rice et al., 2015) was contradictory concerning the presence of a B chromosome in the chromosome set of Aeluropus. The FISH mapping with telomere- and 45S rDNA-specific probes clearly indicated that metaphases where more than 20 chromatin units were counted, are the result of extended NORs with only very thin chromatin fibers between the satellite and the corresponding chromosome. Such decondensed chromosomal rDNA sites were also described for *Lolium* and *Festuca* genotypes (Rocha et al., 2017). When only a DNA stain is applied, distally located satellites can easily be miscounted as separate chromosomes. At least for the plant material analyzed in the presented dataset the occurrence of B-chromosomes can be excluded. Whether the chromosome counts deposited in the Chromosome Counts Database (Rice et al., 2015), where the presence of B chromosomes in this species was reported, are indeed correct remains to be answered. However, in the closely related species *Aeluropus macrostachyus Hack*., the presence of B chromosomes was also described (*x* = 10 + 1B). In contrast, in *Aeluropus lagopoides (L.) Thwaites* (*x* = 10) no B chromosomes were detected (Rice et al., 2015). Therefore, the base chromosome number within the genus is considered stable with x = 10 A chromosomes.

**TABLE 3:** Statistics of *Aleuropus* genome assembly.

		*Oryza sativa*	*Sorghum bicolor*	*Brachypodium distachyon*	*Aeluropus littoralis*
	Assembly	*IRGSP-1.0, INSDC*	*Sorghum_bicolor_NCBIv3*	Brachypodium_distachyon_v3.0	*This study*
Chromosome number	(2n)	24	20	10	20
Genome	Size	500 Mb	730 Mb	355 Mb	354 Mb
Sequencing	covered	375,049,285	675,363,888	270,739,641	300,381,201
Sequenced	%	75	93	76	85
Gene number	annot.	37,849	34,118	34,310	15,916

As indicated in Figure 4 approximately 85% of the genome information is covered by the presented sequencing approach. The number of 15,916 annotated gene models is relatively small, compared to other monocotyledonous plants (Supplementary Table 2). However, a substantial amount of genes has been identified and the sequence information can be used for further research. In addition, the repeat fraction analysis revealed that 21.6% of the genome is composed by different repetitive elements, mainly by tandem repeat sequences distributed in several satellite DNA families (Table 2). The high abundance of the AlSat repeat family makes it likely that this provides a function as centromere building block. Small genomes are known to contain low amounts of repetitive sequences, which furthermore are constituted mainly by tandem repeats, as satellite DNAs, but less transposable elements (Macas et al., 2015). Thus, the repeat composition of *A. littoralis* is in agreement with this assumption of small genome composition.

In monocotyledonous plants the plastid genome is maternally inherited and excluded from sexual recombination. Taking the highly conserved chloroplast genome as proxy for the entire genome it can be stated that our sequencing approach covers preferentially genic regions, while repetitive sequences are not well assembled. The plastid genome also shows that no small genes (such as transfer-RNA genes) are included in the annotation (Supplementary Table 3).

As shown in Figure 2, *A. littoralis* is not only able to survive, but also to grow and develop on high soil salinity (Barhoumi et al., 2007b) and tolerate high salt concentrations in the soil up to 1 M sodium chloride. The plant is able to secrete salt *via* its salt glands leading to formation of salt crystals on the leaf surface (Barhoumi et al., 2007b,^2008^). As reported before, we could also confirm the C_4_ carbon fixation mechanism, based on Kranz anatomy (Figure 2). The ultrastructural analysis indicates an unusual feature the bundle sheath cells seem to be more compact, with an increase in stainable compounds. This might lead to a stronger differentiation of the tissues, and a better separation of the compartments, required for a more effective C_4_ photosynthesis. Also the uptake of salt *via* the roots and formation of salt glands, followed by the secretion of salt is an interesting feature of this plant where the genome data might contribute to molecular insights into developmental and acclimation processes.

Using the generated genomic sequence information it was possible to identify 16 genes encoding motifs structures typical for the DREB-transcription factor family, less than reported for other monocotyledonous plants: 20 in pineapple [*Ananas comosus* (Chai et al., 2020)], 57 in rice [*Oryza sativa* (55)] or 29 in wild sugarcane [*Saccharum spontaneum* (Li et al., 2021)]. Although there is still the chance of missing members of the DREB-transcription factor family, our data demonstrate the usability of the generated genome information. In particular the high number of DREB-transcription factors from subfamily A6 (V) are of particular interest as these might be related to salt induced generation of salt glands, a morphological leaf architecture modification based on salt stress unique to Aeluropus.

We are aware that our genomic study only is a glimpse into the genome of *A. littoralis* and can be complemented with a broader usage of biotechnological methods to reach a more comprehensive picture of this extraordinary species. However, we do show how versatile results can be by using a simple WGS approach and want to share the generated information on the Aleuropus genome sequence.

## Data Availability Statement

The datasets presented in this study can be found in online repositories. The names of the repository/repositories and accession number(s) can be found in the article/Supplementary Material.

## Author Contributions

SH-P, MA, BD, Y-TK, and AH performed experiments. SH-P and MA performed DREB gene family analysis. TS performed bioinformatic genome analysis. MB performed repeat masker study. SH-P, GN, SM, TA, and MK conceptualized the work and wrote the manuscript. All authors contributed to the article and approved the submitted version.

## Conflict of Interest

The authors declare that the research was conducted in the absence of any commercial or financial relationships that could be construed as a potential conflict of interest.

## Publisher’s Note

All claims expressed in this article are solely those of the authors and do not necessarily represent those of their affiliated organizations, or those of the publisher, the editors and the reviewers. Any product that may be evaluated in this article, or claim that may be made by its manufacturer, is not guaranteed or endorsed by the publisher.

## Footnotes

1
http://www.bioinformatics.babraham.ac.uk/projects/fastqc/

2
https://github.com/groupschoof/AHRD/

3
http://rice.plantbiology.msu.edu/

4
https://www.arabidopsis.org/

5
https://pfam.xfam.org/

6
https://www.ebi.ac.uk/interpro/

7
http://smart.embl-heidelberg.de/

8
https://web.expasy.org/protparam/

9
https://meme-suite.org/meme/db/motifs

## Supplementary Material

Supplementary Figure 1 Bar diagram indicating the actual available sequence information for O. sativa, S. bicolor, *B. distachyon* and *A. littoralis.* Detailed number and data sources are supplemented in Supplementary Table 1.

Supplementary Figure 2 Phylogenetic structure of *Aeluropus littoralis* DREB-proteins. Phylogenetic comparison *of Aeluropus littoralis* DREB-proteins (blue circles) with Oryza sativa and *Arabidopsis thaliana* DREB-proteins. Individual Subfamilies (Sakuma et al., 2002) A1-A6 (I-V) are indicated and *Aeluropus littoralis* DREB-proteins are indicated by blue circles. (The respective AA sequence of the proteins and motifs can be found in Supplementary Table 4).

Supplementary Table 1 Protocol for assisted chemical fixation, substitution and resin embedding for microscopy.

Supplementary Table 2 Comparison of Aeluropus genome assembly with rice, millet and stiff brome.

Supplementary Table 3 Comparison of predicted genes.

Supplementary Table 4 Sequence information of DREB-genes.

Supplementary Table 5 Sequence information for DEB motifs.

## Abbreviations

BS: bundle sheath
DREB: dehydration responsive element-binding
ESTs: expressed sequence tag
FISH: fluorescence *in situ* hybridization
Mbp: megabasepairs
M: mesophyll
NOR: nucleolus organizing region
PE: paired end reads
satDNA: satellite DNA
WGS: whole-genome sequencing approach.
