# Supplementary figures and images for: Initial Description of the Genome of Aeluropus littoralis, a Halophile Grass
Source: Front Plant Sci. 2022 Jul 11;13:906462. doi: 10.3389/fpls.2022.906462 (PMC9310549; doi:10.3389/fpls.2022.906462)

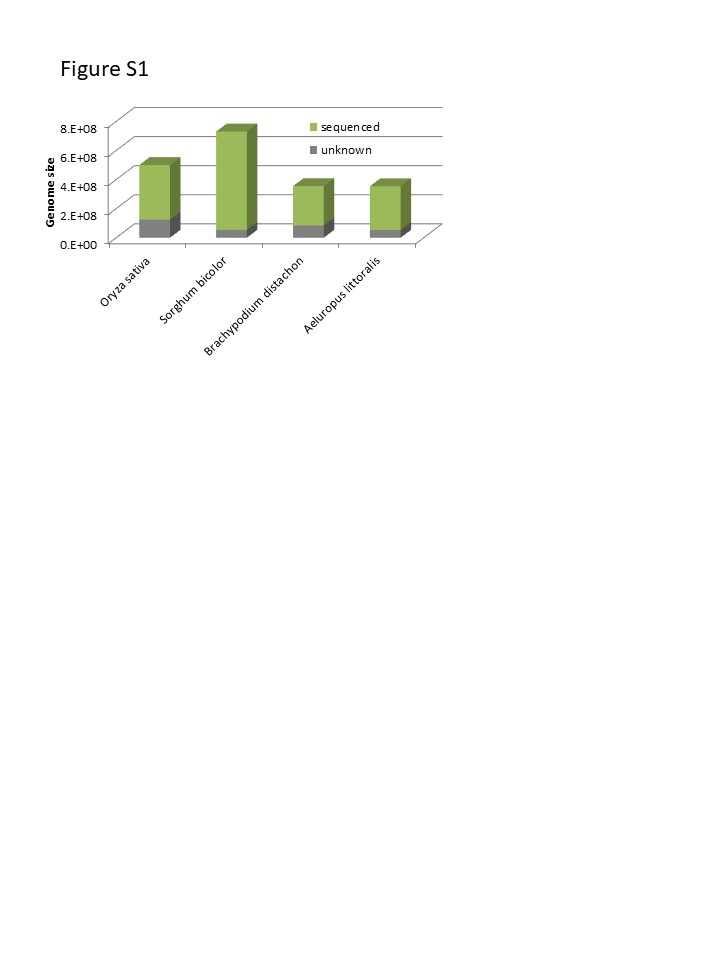

Supplement: Supplementary Figure 1 — Bar diagram indicating the actual available sequence information for O. sativa, S. bicolor, B. distachyon and A. littoralis. Detailed number and data sources are supplemented in Supplementary Table 1. [file Image_1.JPEG]

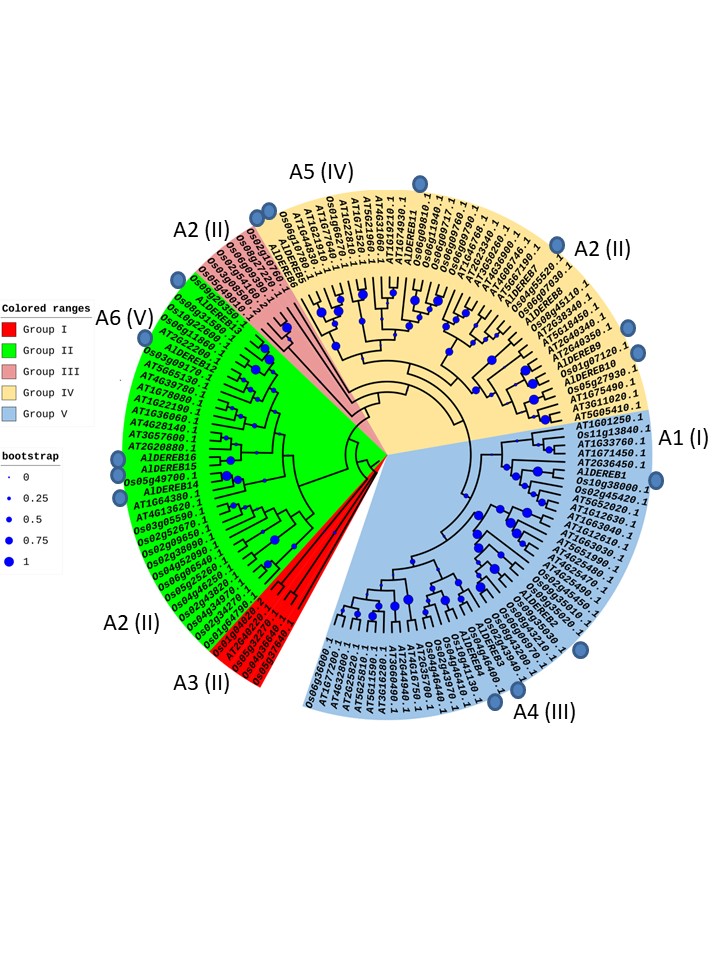

Supplement: Supplementary Figure 2 — Phylogenetic structure of Aeluropus littoralis DREB-proteins. Phylogenetic comparison of Aeluropus littoralis DREB-proteins (blue circles) with Oryza sativa and Arabidopsis thaliana DREB-proteins. Individual Subfamilies (Sakuma et al., 2002) A1-A6 (I-V) are indicated and Aeluropus littoralis DREB-proteins are indicated by blue circles. (The respective AA sequence of the proteins and motifs can be found in Supplementary Table 4). [file Image_2.JPEG]
