# Supplementary material for: Initial Description of the Genome of Aeluropus littoralis, a Halophile Grass
Source: Front Plant Sci. 2022 Jul 11;13:906462. doi: 10.3389/fpls.2022.906462 (PMC9310549; doi:10.3389/fpls.2022.906462)
Supplement: Supplementary Table 1 — Protocol for assisted chemical fixation, substitution and resin embedding for microscopy. [file Table_1.DOCX]

**Supplemental Table 1:** Protocol for combined conventional and microwave-proceeded chemical fixation, dehydration and embedding in Spurr resin of *Aeluropus* leaf cuttings for histological and ultrastructural analysis.

| **Combined conventional chemical fixation & micowave processing in a**  **PELCO Bio Wave®34700-230 (Ted Pella, Inc., Redding CA, USA)** | | | | |
| --- | --- | --- | --- | --- |
| **Process** | **Reagent** | **Power**  **[W]** | **Time**  **[sec]** | **Vacuum**  **[mm Hg]** |
| **1. Primary**  **fixation** | 2.0% (v/v) glutaraldehyde  and 2.0% (v/v) paraformaldehyde  in 0.05 M cacodylate buffer (pH 7.3) | 0  150  0  150 | 60  60  60  60 | 0  0  0  0 |
|  | additional 30 minutes on a shaker at room temperature | | | |
| **2. Wash** | 1x 0.05 M cacodylate buffer (pH 7.3 ) and 2x aqua dest. | 150 | 45 | 0 |
| **3. Secondary**  **fixation** | 1% (v/v) osmiumtetroxide  in aqua dest. | 0  100  0  100  0  100 | 60  120  60  120  60  120 | 10  10  10  10  10  10 |
| **4. Wash** | 3x aqua dest. | 150 | 45 | 0 |
| **5. Dehydration** | Acetone:30%, 40%, 50%, 60%,  70%, 80%, 90%, 1x 100%  and 1 x propylenoxide. | 150 | 45 | 0 |
|  | after each step samples were kept for additional 5 min on a shaker | | | |
|  | 25% Spurr resin in propylenoxide | 2 hrs on shaker at RT | | |
| **6. Resin Infiltration** | 50% Spurr resin in propylenoxide | 2 hrs on shaker at RT | | |
|  | 75% Spurr resin in propylenoxide | 2 hrs on shaker at RT | | |
|  | 100% Spurr resin | over night on shaker at RT | | |
| **7. Polymerisation** | 24 hrs at 70°C in flat embedding moulds in a heating cabinet. | | | |
